# Tillaux Fracture in an Adolescent Patient: A Case Report

**DOI:** 10.7759/cureus.98994

**Published:** 2025-12-11

**Authors:** María Mercedes Medina Villate, Alberto Daniel Navarro Vergara

**Affiliations:** 1 Orthopedics and Traumatology, Hospital de Especialidades Quirúrgicas Ingavi, Instituto de Previsión Social, Asunción, PRY; 2 Orthopedics and Traumatology, Hospital de Trauma Prof Dr Manuel Giani, Asunción, PRY; 3 Orthopedics and Traumatology, Instituto de Previsión Social, Asunción, PRY

**Keywords:** adolescent tibial fracture, aofas scale, closed reduction percutaneous fixation, salter harris iv, tillaux fracture

## Abstract

Tillaux fractures are infrequent adolescent fractures that are rarely covered in the clinical literature. They are intra-articular fractures that are more frequent in children than adults, more commonly seen in women. The treatment is operative, and the mini-open technique is a technique that we should consider using. This article presents the case of a 14-year-old girl who suffered a fall from height with twisting trauma to her left ankle. On physical examination, the affected limb showed pain and functional limitation, with palpable distal pulses, adequate capillary refill, and no motor or sensory deficits. An X-ray was made, and a Tillaux fracture was found. She was immediately immobilized with a splint, and once the CT images were obtained, surgical management was planned. An operative approach was selected, consisting of closed reduction and percutaneous fixation with a single cannulated screw. It is essential for orthopedic surgeons to accurately recognize this type of fracture to ensure appropriate diagnosis and optimal treatment outcomes. The article highlights the importance of surgical intervention in these rare fractures in a specific age range and demonstrates the favorable outcomes of surgical treatment using a percutaneous technique. It also emphasizes that early presentation to the hospital plays a crucial role in minimizing the risk of complications.

## Introduction

Ankle injuries are a common reason for presentation to the hospital, the American Orthopedic Foot and Ankle Society (AOFAS) score is one of the most frequently used scores for foot and ankle conditions [1]. Tillaux fractures are intra-articular fractures that affect the physis and epiphysis of the distal tibia and are commonly observed in children and adolescents [2]. The epidemiology indicates that this fracture accounts for approximately 3-5% of pediatric ankle fractures in patients aged 12 to 14 years and is reported to occur more commonly in females [3]. A few cases have also been described in adults, but other investigators have reported this to be an uncommon finding in that population [4]. The symptoms typically include pain and swelling in the anterolateral part of the ankle. Currently, several classification systems exist, but the Dias-Tachdjian classification is considered useful for both triplanar and Tillaux fractures [5].

A systematic review found that surgery for Tillaux fractures is usually recommended when the fracture is displaced by more than 1-2 mm [6]. Multiple treatment approaches have been described for managing these fractures, though none have shown superior outcomes over the others [7].

## Case presentation

A 14-year-old female patient presented to the hospital with a story of a fall from height during recreational activity without loss of consciousness, with twisting trauma on the left ankle. The physical examination revealed the following: pain and functional impairment in the affected limb, palpable distal pulses, good capillary filling, and no motor or sensory deficit.

**Figure 1:**
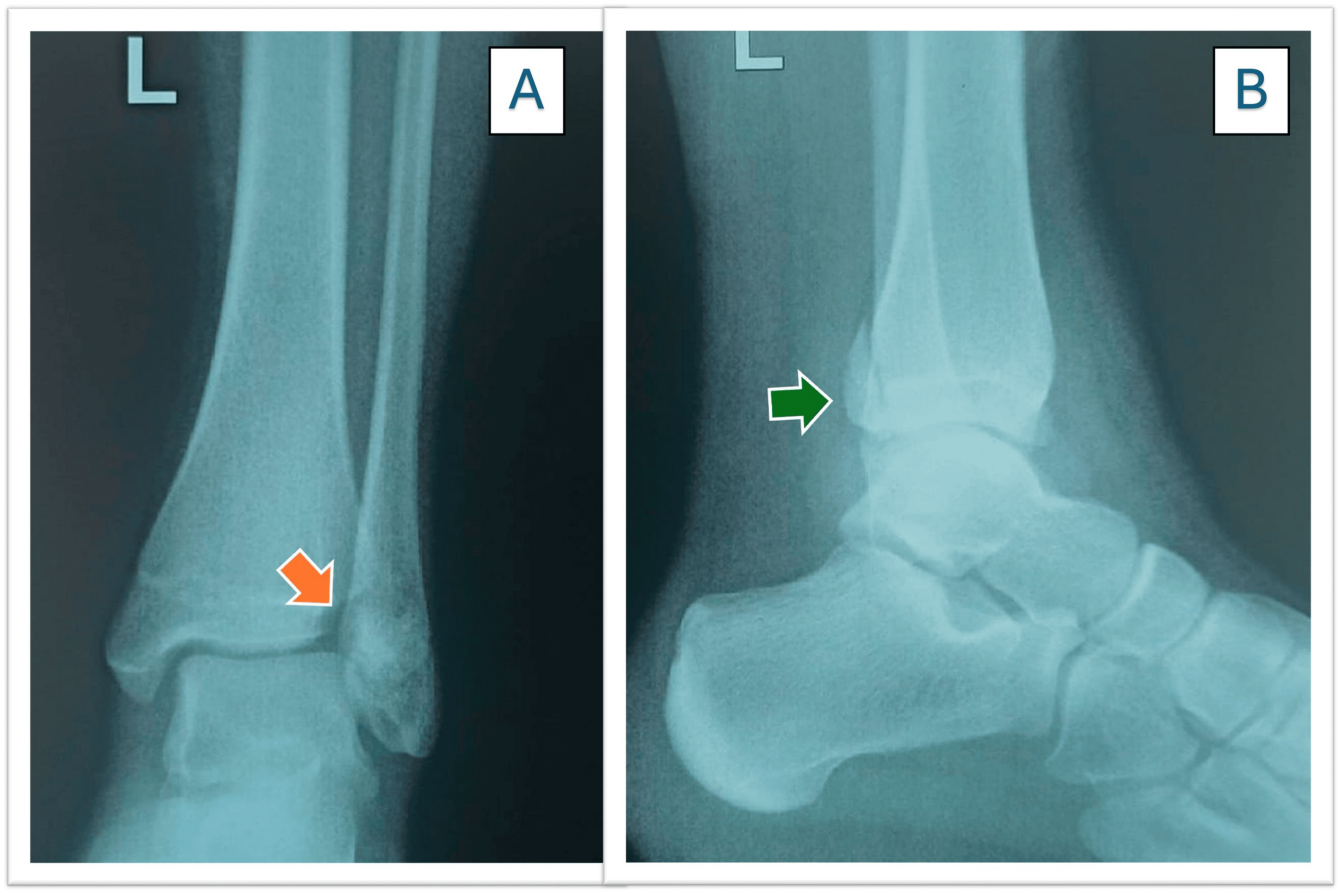
Tillaux fracture X-ray. A) Anteroposterior view, and the arrow (orange) shows the fracture. B) Lateral view, and the arrow (green) shows the fracture.

Therefore, portable imaging studies were requested. The radiographs revealed a Tillaux fracture in the distal left tibia, and a Salter-Harris type IV (SH IV) injury was confirmed (Figure 1). Additionally, the lateral view demonstrated a posterior malleolus fracture, indicating that this was not an isolated Tillaux fracture.

**Figure 2:**
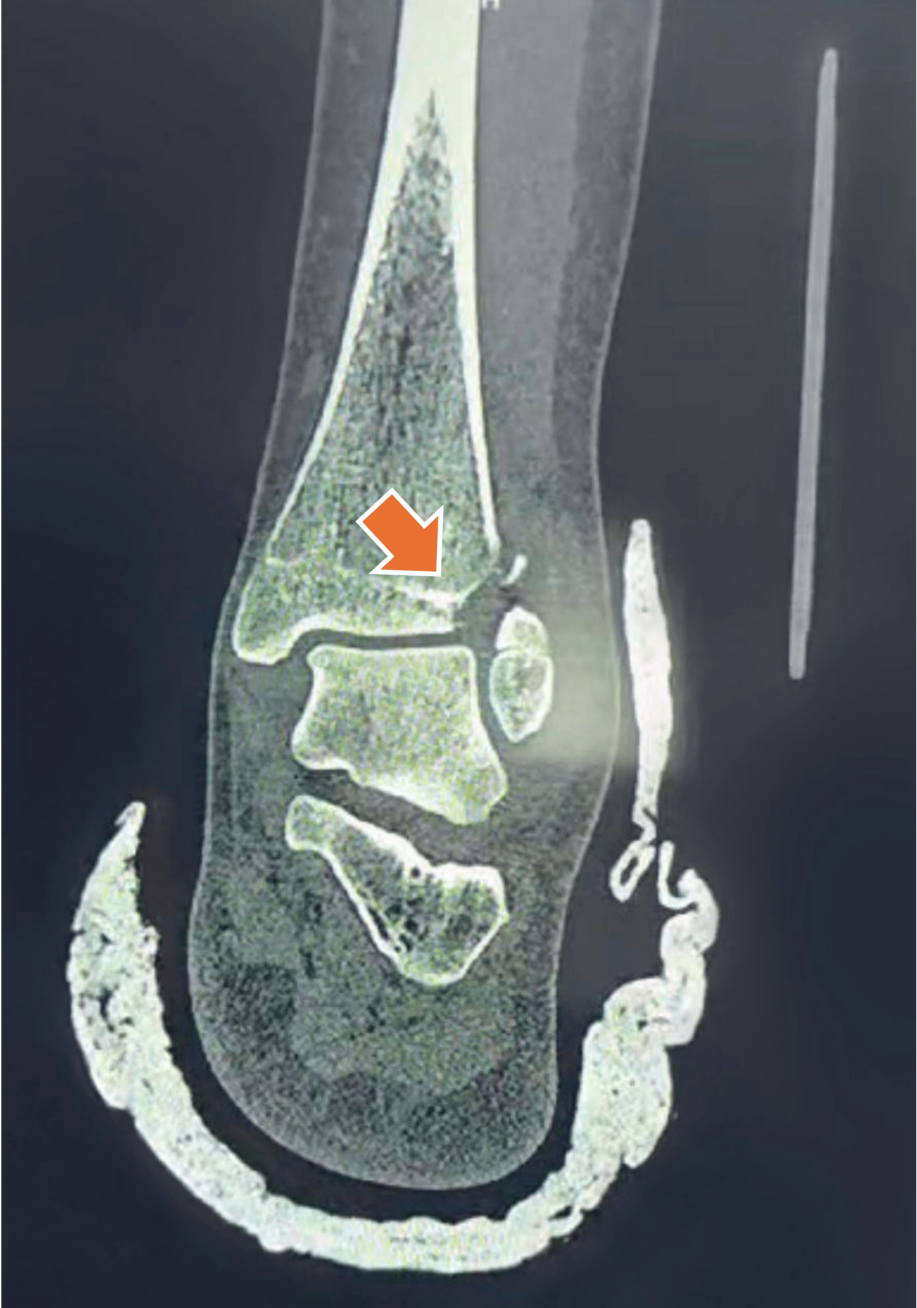
Tillaux’s fracture coronal cut. The orange arrow shows the fracture traces.

**Figure 3:**
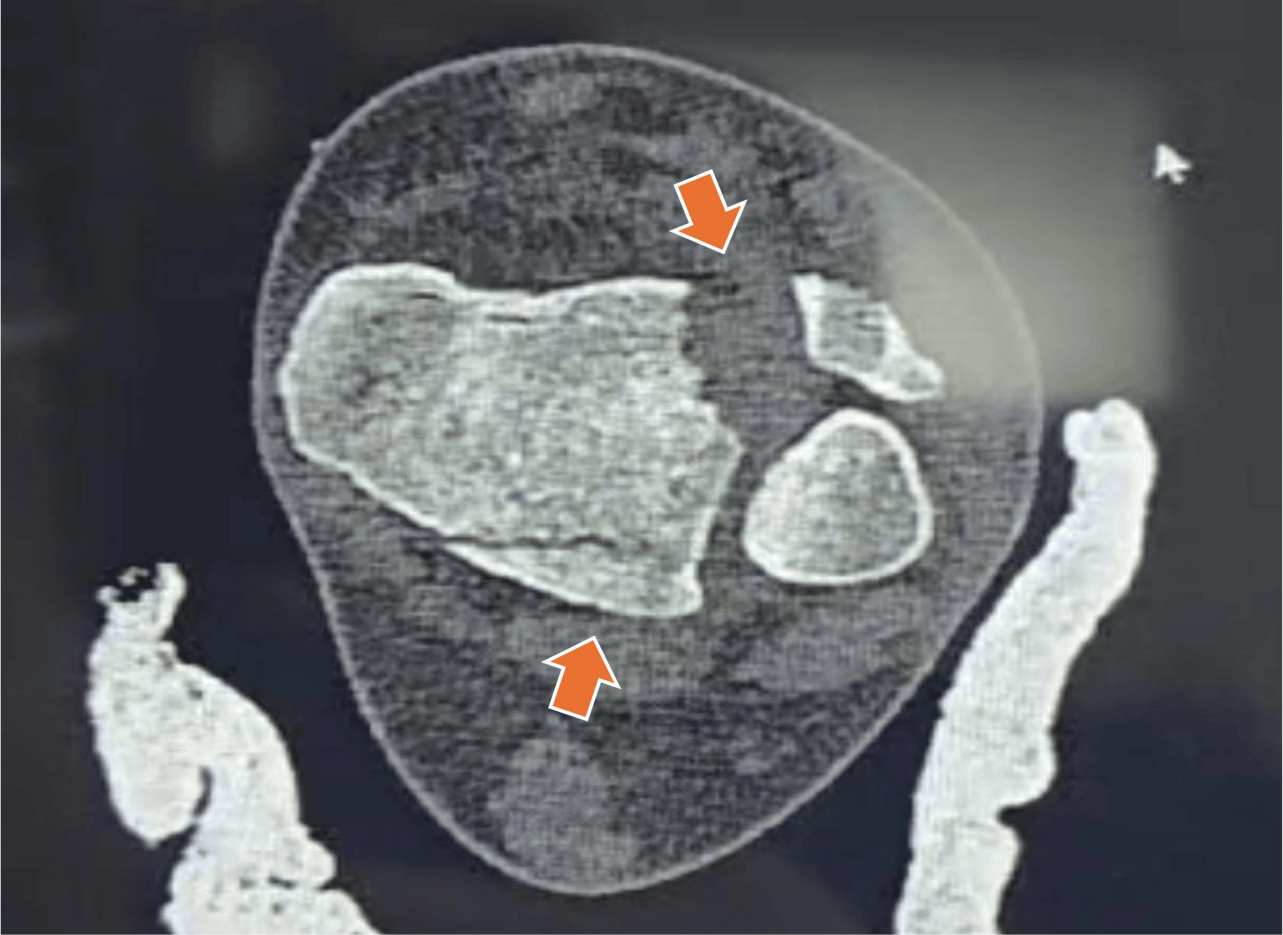
Tillaux’s fracture axial cut. The orange arrows show fracture traces.

**Figure 4:**
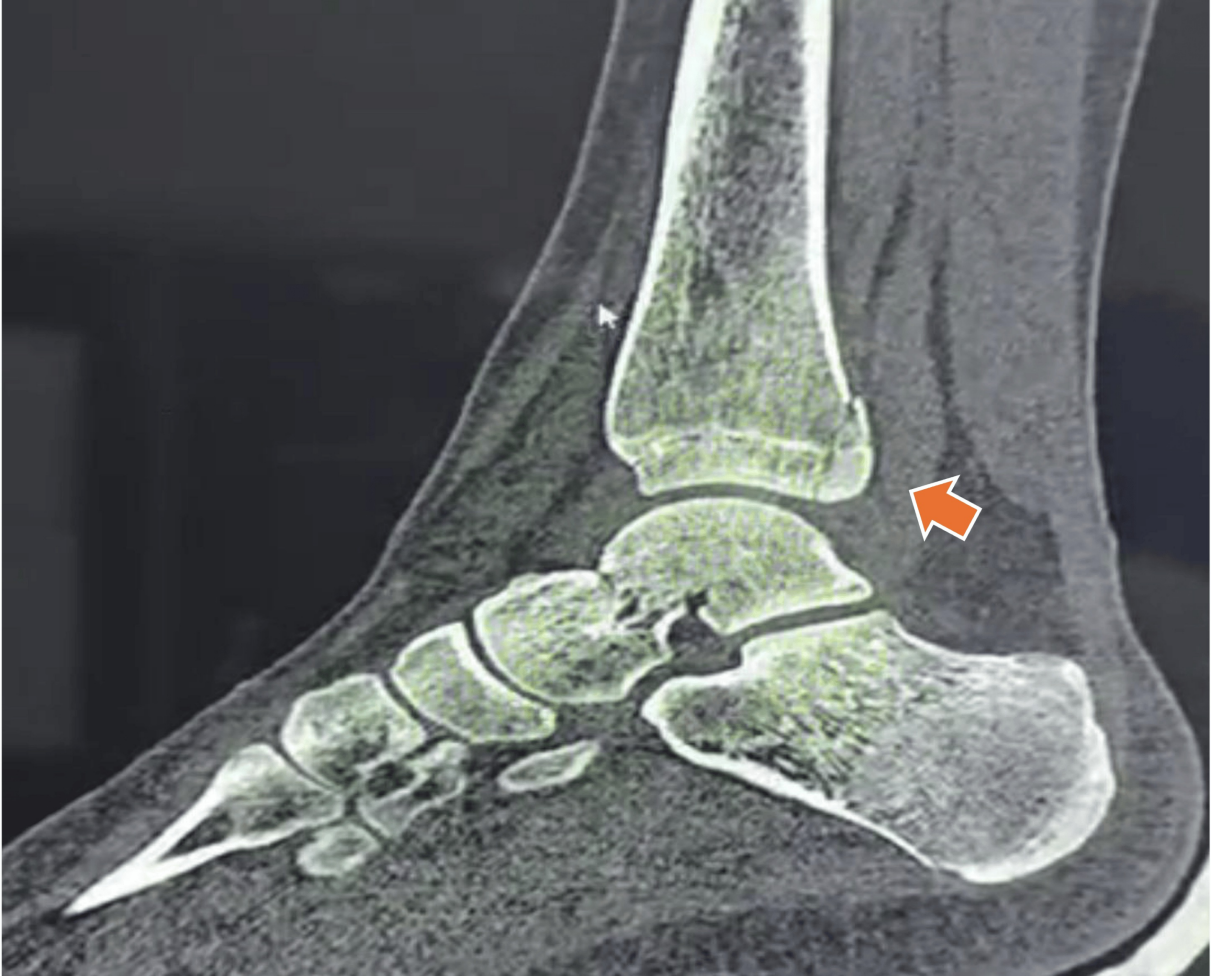
Posterior malleolus fracture, sagittal CT. The orange arrow indicates the posterior malleolus fracture line. The Tillaux fragment is located anteriorly, as demonstrated on the axial CT.

The patient was immediately immobilized with a splint, and tomographic images were requested to plan the surgery (Figures 2-4).

The performed surgery was a closed reduction percutaneous fixation (CRPF). Reduction was achieved under fluoroscopic guidance using gentle longitudinal traction and internal rotation of the foot, followed by percutaneous placement of a cannulated screw with a washer across the epiphysis, parallel to the articular surface.

**Figure 5:**
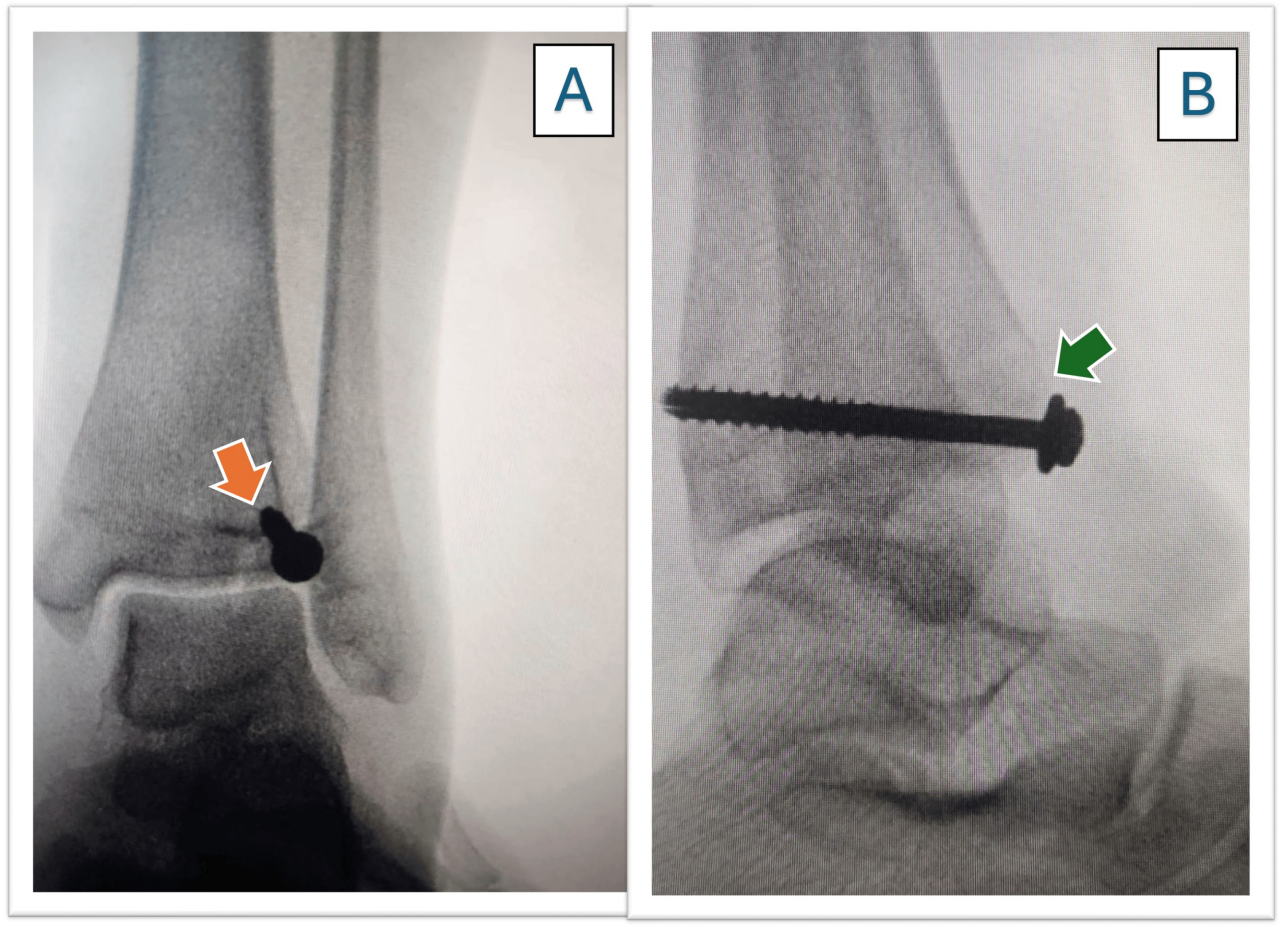
Anterioposterior and lateral postoperative radiographs. A) The orange arrow indicates the position of the cannulated screw in the anteroposterior view, showing an acceptable reduction with a minimal residual articular gap. B) the green arrow shows the lateral view, where the alignment is satisfactory despite the presence of a small articular gap.

The patient left the operating room with a splint for the first week and remained non-weight-bearing for four weeks while performing isometric exercises. At follow-up, the patient returned with improved clinical status, and the follow-up X-rays showed favorable healing progress (Figures 5A, 5B).

Kinesic rehabilitation was not suggested because it would not make a difference in the evolution. Recreational and daily activities at home were permitted, along with isometric exercises as previously instructed. The patient was advised strict follow up regularly to assess ankle range of motion and muscle strength over the next three months.

**Figure 6:**
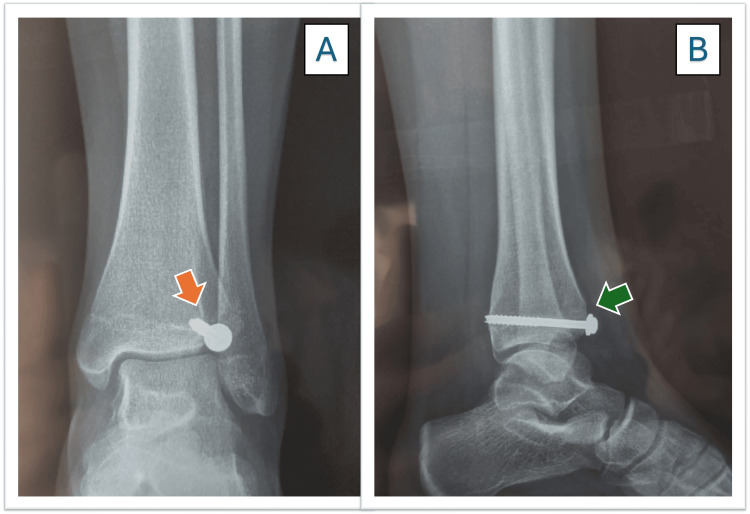
Anteroposterior and lateral view radiographs three months after surgery. A) The orange arrow shows a correct consolidation in the anterior-posterior view. B) The green arrow shows a correct consolidation in the lateral view.

At the two-month follow-up visit, the patient came in using crutches for support. His ankle mobility had improved well, though the range of motion was still diminished. At the periodical check-ups at the third month, signs of consolidation were identified on the X-ray (Figures 6A, 6B).

During the evaluation, in the follow-up visits, the patient obtained 92 points on the AOFAS scale at 75 days post-surgery.

**Figure 7:**
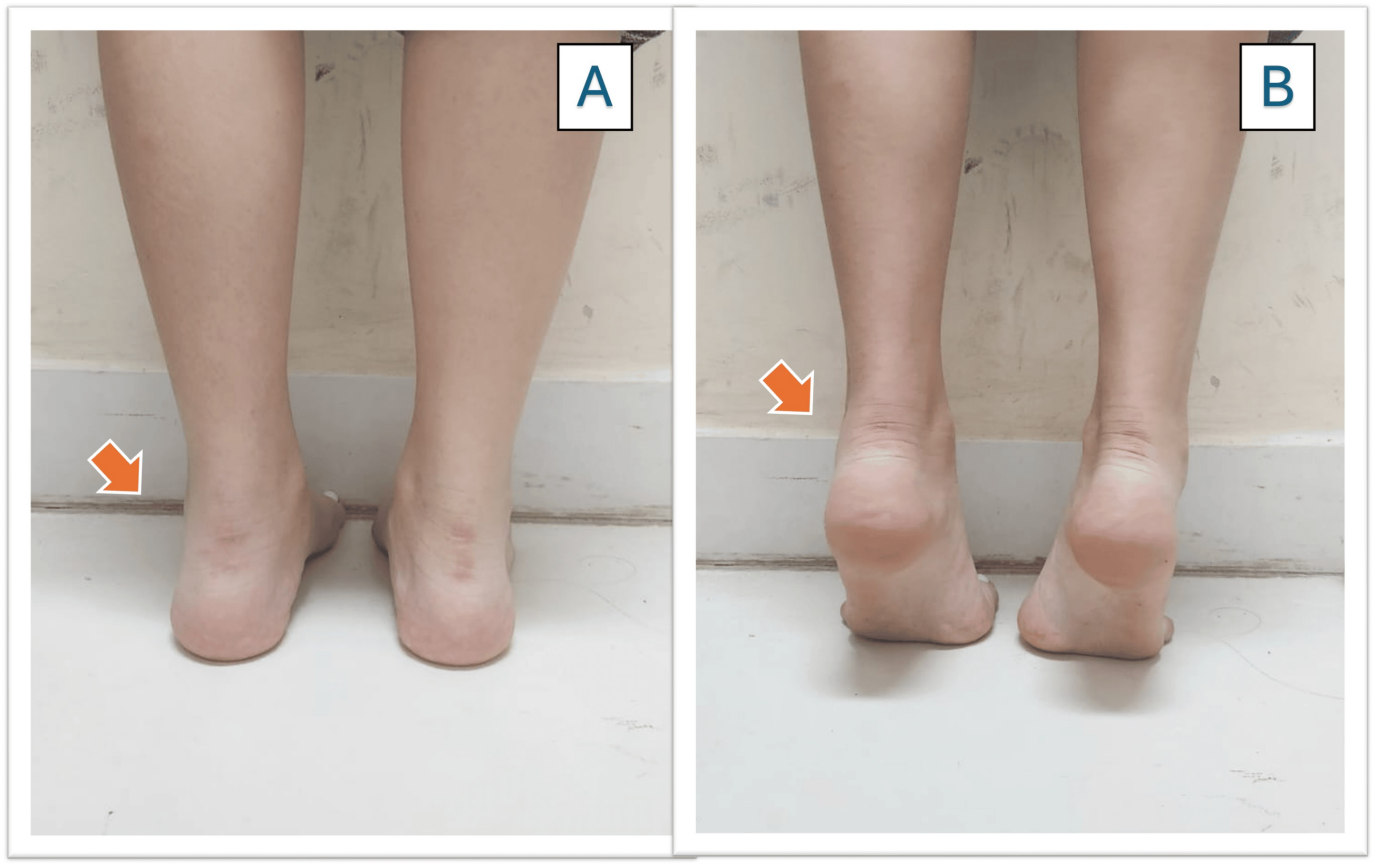
Posterior view of the ankle. A) Good angulation of the ankle and foot without consequent deformities. B) Good joint range with good muscle strength.

At the third month’s appointment, during the physical examination, the patient entered on her own, without support, without pain, with normal gait and good angulation of the ankle and foot, without deformities resulting from the fracture (Figure 7A). The ankle demonstrated good joint range of motion and muscle strength, with no signs of swelling on examination (Figure 7B).

The patient had an improvement in the AOFAS scale at 90 days post-surgery, reaching a total of 100 points.

## Discussion

Giacobazzi et al. referred to Salter-Harris (SH) as a classification for the physis. Salter-Harris type IV fractures are intra-articular injuries in which the fracture line extends from the epiphysis, crosses the physis, and continues into the metaphysis [8]. Tillaux fractures are rare fractures that occur in adolescent age groups [8]. Furthermore, epidemiological factors relevant to adolescent Tillaux fractures were reviewed. Adult ankle fracture mechanisms, such as pronation-external rotation patterns, do not apply to pediatric injuries and are not included here, as pediatric and adolescent ankle fractures are more accurately classified using the Dias-Tachdjian system [9]. Additionally, the literature on this topic is limited, making it an interesting case for further investigation.

Tillaux fractures refer to the avulsion fracture of the anterior tibiofibular ligament at the starting point of the tibia [10]. It is commonly seen in 12-year-old patients referred by most studies [11], but it was also mentioned to be seen in seven adult patients. The study was published on PubMed (2006-2020) [12]. Recently, another study, which included a transgender person, was also published; it required surgical treatment [13]. In addition, another study said that the most common cause was ankle sprain [14].

Several other studies were conducted, including a child who had a Maisonneuve fracture associated with the Tillaux fracture, and whose treatment was operative [15]. More recently, a study suggested that computed tomography with 3D analysis could be a valuable tool in such cases [16].

Regarding displaced Tillaux fractures, the literature indicates that treatment may involve either closed or open fixation. In all cases, careful follow-up is required [17]. Recently, other researchers referred to their technique as open reduction and fixation with double screw [18]. Another study reported regarding the technique used, there were no significant differences between epiphyseal and trans-epiphyseal screws [19]. In addition, the use of washers should be considered by professionals, considering previous cases like Giacobazzi (SH III) [18]. The literature reports that it should be considered to use the mini-open technique, which has many benefits [20]. A review of surgical outcomes for Tillaux fractures indicates good prognoses, with a mean score of 85.6±7.9 over follow-up periods ranging from 18 to 70 months, as documented by Wei et al. [21]. Orthopedic practitioners should use standardized scoring systems during follow-up to assess long-term results.

## Conclusions

Tillaux fractures are uncommon and occur more frequently in children than in adults. In adolescents, they are rare. For this reason, careful evaluation of ankle injuries is essential in order to identify atypical fracture patterns and ensure appropriate treatment. Early and accurate diagnosis helps prevent complications and supports optimal functional recovery.
